# Scaling up task-sharing psychological interventions for refugees in Jordan: a qualitative study on the potential barriers and facilitators

**DOI:** 10.1093/heapol/czad003

**Published:** 2023-01-12

**Authors:** Aniek Woodward, Egbert Sondorp, Alexandra S Barry, Marjolein A Dieleman, Daniela C Fuhr, Jacqueline E W Broerse, Aemal Akhtar, Manar Awwad, Ahmad Bawaneh, Richard Bryant, Marit Sijbrandij, Pim Cuijpers, Bayard Roberts

**Affiliations:** KIT Royal Tropical Institute, KIT Health, Mauritskade 64, Amsterdam 1092 AD, The Netherlands; Athena Institute, Amsterdam Public Health Research Institute, Vrije Universiteit Amsterdam, De Boelelaan 1085, Amsterdam 1081 HV, The Netherlands; KIT Royal Tropical Institute, KIT Health, Mauritskade 64, Amsterdam 1092 AD, The Netherlands; KIT Royal Tropical Institute, KIT Health, Mauritskade 64, Amsterdam 1092 AD, The Netherlands; NHS England, 133-155 Waterloo Road, London SE1 8UG, UK; KIT Royal Tropical Institute, KIT Health, Mauritskade 64, Amsterdam 1092 AD, The Netherlands; Athena Institute, Amsterdam Public Health Research Institute, Vrije Universiteit Amsterdam, De Boelelaan 1085, Amsterdam 1081 HV, The Netherlands; Department of Health Services Research and Policy, Faculty of Public Health and Policy, London School of Hygiene and Tropical Medicine, Keppel Street, London WC1E 7HT, UK; Department of Prevention and Evaluation, Leibniz Institute for Prevention Research and Epidemiology-BIPS, Achterstraße 30, Bremen 28359, Germany; University of Bremen, Health Sciences, Bibliothekstrasse 1, Bremen 28359, Germany; Athena Institute, Amsterdam Public Health Research Institute, Vrije Universiteit Amsterdam, De Boelelaan 1085, Amsterdam 1081 HV, The Netherlands; School of Psychology, University of New South Wales, Kensington, Sydney NSW 2052, Australia; Division of Insurance Medicine, Department of Clinical Neuroscience, Karolinska Institutet, Solnavägen 1, Solna 171 77, Sweden; International Medical Corps, Al Shareef Abd Al Hameed Sharaf St 9, Amman, Jordan; International Medical Corps, Al Shareef Abd Al Hameed Sharaf St 9, Amman, Jordan; School of Psychology, University of New South Wales, Kensington, Sydney NSW 2052, Australia; Department of Clinical, Neuro and Developmental Psychology, World Health Organization Collaborating Center for Research and Dissemination of Psychological Interventions, Amsterdam Public Health Research Institute, Vrije Universiteit Amsterdam, De Boelelaan 1105, Amsterdam 1081 HV, The Netherlands; Department of Clinical, Neuro and Developmental Psychology, World Health Organization Collaborating Center for Research and Dissemination of Psychological Interventions, Amsterdam Public Health Research Institute, Vrije Universiteit Amsterdam, De Boelelaan 1105, Amsterdam 1081 HV, The Netherlands; International Institute for Psychotherapy, Babeș-Bolyai University, 37 Republicii Street, Cluj-Napoca, Romania; Department of Health Services Research and Policy, Faculty of Public Health and Policy, London School of Hygiene and Tropical Medicine, Keppel Street, London WC1E 7HT, UK

**Keywords:** Refugees, Jordan, Syria, mental health, implementation, research to policy, health systems research, qualitative research

## Abstract

Training nonspecialists in providing evidence-based psychological interventions (i.e. task-sharing) can effectively increase community access to psychological support. However, task-sharing interventions for this purpose are rarely used at scale. The aim of this study was to examine the factors influencing the potential for scaling up (i.e. scalability) of a task-sharing psychological intervention called Problem Management Plus (PM+) for Syrian refugees in Jordan. Semi-structured individual (*n* = 17) and group interviews (*n* = 20) were conducted with stakeholders knowledgeable about PM+ and the mental health system for Syrian refugees in Jordan. Using ‘system innovation perspective’, this study conceptualized the context as landscape developments, and systemic considerations were divided into culture (shared ways of thinking) and structure (ways of organizing). Political momentum was identified as a landscape trend likely facilitating scaling up, while predicted reductions in financial aid was regarded as a constraint. In terms of culture, the medicalized approach to mental health, stigma and gender were reported barriers for scaling up PM+. Using non-stigmatizing language and offering different modalities, childcare options and sessions outside of working hours were suggestions to reduce stigma, accommodate individual preferences and increase the demand for PM+. In relation to structure, the feasibility of scaling up PM+ largely depends on the ability to overcome legal barriers, limitations in human and financial resources and organizational challenges. We recommend sustainable funding to be made available for staff, training, supervision, infrastructure, coordination, expansion and evaluation of ‘actual’ scaling up of PM+. Future research may examine the local feasibility of various funding, training and supervision models. Lessons learned from actual scaling up of PM+ and similar task-sharing approaches need to be widely shared.

Key messages

- Task-sharing approaches as used in the psychological intervention Problem Management Plus (PM+) can effectively increase access to psychological support for communities with high mental health needs, such as refugees, but are to date rarely used at scale.
- We used a system innovation perspective to identify contextual and systemic factors influencing the scaling up of PM+ among Syrian refugees in Jordan.
- Our study foresees limitations in human and financial resources as major barriers in sustainably integrating task-sharing interventions in existing systems.
- Scalability considerations and recommendations discussed in this paper may guide future scale up of task-sharing approaches for refugee mental health in Jordan and similar resource-constraint contexts.

## Introduction

Transformation in the implementation of mental health care is urgently needed, particularly in light of ongoing global threats to mental health such as growing social inequalities, protracted conflicts and public health emergencies (Patel *et al.*, 2018; WHO, 2022b). Progress in global mental health has been slow and consequently “too many people living with mental health conditions are not getting the care they need and deserve” (WHO, 2022b). Around a billion people worldwide are suffering from mental disorders, of which 80% reside in low- and middle-income countries (LMICs) (IHME, 2019). Mental health resources are particularly limited in LMICs (WHO, 2021), resulting in a large treatment gap. For example, only 1 in 27 people with depression living in LMICs receive adequate treatment (compared with 1 in 5 in high-income countries) (Thornicroft *et al.*, 2017).

Task-sharing between specialist and nonspecialist mental health providers is considered an innovative solution to the unsustainable situation in global mental health (Patel *et al.*, 2018; WHO, 2022b). Task-sharing can strengthen the workforce and enhance access to psychological care, e.g. by training primary healthcare providers or community workers in providing ‘simple’ evidence-based psychological interventions under supervision of a mental health professional. This approach is commonly used in LMICs and humanitarian settings, with increasing evidence on the effectiveness of training nonspecialists and its benefits to the mental health and quality of life of beneficiaries (Bangpan *et al.*, 2019; Caulfield *et al.*, 2019; Murray *et al.*, 2019; Van Ginneken *et al.*, 2021). Despite their potential, until now, task-sharing psychological interventions have rarely been successfully implemented at scale (Cohen and Yaeger, 2021; Patel, 2022). An explanation is that ‘scaling up’ task-sharing requires a shift towards community-based care and stepped-care models, which requires restructuring of the existing health system.

‘Scaling up’ can be defined as the process of integrating innovative interventions into existing systems (Simmons and Shiffman, 2007). Previous research reported numerous barriers in the implementation and integration of task-sharing and community-based approaches in mental health, such as complexity of service users’ needs, low acceptability by beneficiaries and other providers, insufficient human and financial resources, lack of facilities and services and inadequate social and mental health policies (Mendenhall *et al.*, 2014; Vandewalle *et al.*, 2016; Chibanda, 2018; Esponda *et al.*, 2020; Shalaby and Agyapong, 2020; Troup *et al.*, 2021). While the barriers are often at health system level, systems thinking to investigate scaling up is underutilized in task-sharing for mental health research (Javadi *et al.*, 2017). This paper applies a ‘system innovation perspective’ with the aim to examine the factors influencing the potential for scaling up (i.e. ‘scalability’), a novel task-sharing psychological intervention called Problem Management Plus (PM+) in Jordan.

Our conceptual framework of a ‘system innovation perspective’ is detailed elsewhere (Woodward *et al.*, 2021). In essence, this perspective considers scaling up to be affected by complex interactions at three embedded levels: (1) ‘niches’ (i.e. protected experimental environments), (2) ‘dominant constellations’ (i.e. cultures, structures and practices of existing (sub)systems) and (3) ‘landscape’ (i.e. broader context) (Geels, 2002). Moving novel approaches proven effective in a research environment to platforms of care requires some level of change in old ways of thinking (‘culture’), organizing (‘structure’), and doing (‘practices’) (Van Raak, 2010). In other words, system change is needed for the scaling up of innovations to be effective and sustainable (Broerse and Grin, 2017). Both culture and structure are structuring elements: shaped by the practices of ‘actors’ (i.e. individuals/groups acting as unity) but also limit what actors can or want to do (Van Raak, 2010) and therefore play an important influencing role in scaling up.

## Methods

### Study setting and context

This study is part of the Syrian REfuGees MeNTal HealTH Care Systems (STRENGTHS) project. STRENGTHS aims to strengthen mental health systems by examining the effectiveness, cost-effectiveness, and scalability of different novel psychological interventions in eight countries hosting Syrian refugees in Europe and the Middle East (Sijbrandij *et al.*, 2017). This study reports on the scalability of group PM+ in Jordan, which was conducted alongside STRENGTHS’ randomized controlled trials (RCTs) on PM+ effectiveness. While the RCTs focused on adult Syrian refugees in a camp setting, in this study, we explored scaling up PM+ more broadly. The primary focus was on Syrian refugees, but we also explored expansion to, e.g. other target groups (e.g. Syrian adolescents, refugees of other nationalities and host population) and in other settings (e.g. urban/non-camp) and intervention modality (e.g. online).

Details about PM+ (Dawson *et al.*, 2015; WHO, 2016) and the Jordanian PM+ pilot and definitive RCTs are published elsewhere (Akhtar *et al.*, 2020; 2021; Bryant *et al.*, 2022a). In brief, PM+ was developed by the WHO and is designed for adults with common mental health symptoms (e.g. depression, anxiety, posttraumatic stress and grief). The intervention consists of five sessions involving psychoeducation, problem-solving techniques and behavioural activation. PM+ is intended as a task-sharing intervention to be provided by nonspecialists following generic manuals that are culturally and locally adapted (Dawson *et al.*, 2015; WHO, 2016). In the Jordanian RCTs, PM+ was conducted by two facilitators in group sessions (∼120 min each), separately for men and women. Facilitators were Jordanian and had a bachelor’s degree in psychology or related health field. Facilitators were trained and supervised in PM+ by a local supervisor, who was trained and supported by an international PM+ master trainer (Akhtar *et al.*, 2020). Results of the trials indicate that it is feasible to train nonspecialists in PM+ and that this can significantly benefit the mental health of refugees (Akhtar *et al.*, 2021; Bryant *et al.*, 2022a).

Jordan—a lower–middle-income, predominantly Muslim and Arabic-speaking country—has the second highest share of refugees per capita globally (Reliefweb 2021). There are currently over 675,000 registered Syrian refugees (UNHCR, 2022) although the total estimates are 1.4 million (Jordan Ministry of Planning and International Cooperation, 2020). About one-fifth of registered Syrian refugees reside in camps, while most are dispersed in urban, peri-urban and rural areas (UNHCR, 2022). The Ministry of Planning and International Cooperation leads the Jordan Response Plan (JRP) of the Syrian refugee crisis at the national level, and the Ministry of Health (MoH) oversees the health component of the response (Ministry of Planning and International Cooperation, 2017). A mapping exercise outlined 33 organizations collectively delivering mental health and psychosocial support (MHPSS) services in Jordan, including the MoH and local and international agencies (IMC, 2021). International Medical Corps is a major MHPSS provider for Syrian refugees in Jordan and delivers its integrated mental health services through comprehensive community clinics inside the camps and through MoH clinics in urban settings. Inside camps, services to Syrian refugees are solely provided by non-governmental organizations (NGOs) and United Nations (UN) agencies, and in urban areas, services are predominantly provided by governmental health providers (sometimes in collaboration with NGOs). UN High Commissioner for Refugees facilities are accessible to refugees at no cost, and the majority (89%) of NGO services are also accessible without cost (IMC, 2016). Since 2019, refugees can make use of MoH-run health services at the rates of non-insured Jordanians (UNHCR, 2021). Still access barriers remain in the public health sector, including ‘unaffordability of services for vulnerable refugees and lack of adequate awareness about the subsidy policy on both the supply and demand sides’ (p. 45) (Samuel Hall & UNHCR Jordan, 2022).

Previous studies in Jordan reported high proportions of adult and under-age Syrian refugees showing signs of psychological distress and other common mental health symptoms like depression and anxiety (IMC and SIGI-JO, 2015; IMC, 2017; Dehnel *et al.*, 2022). Another study showed that Syrian refugees living in urban settings were more likely to experience distress and less likely to have access to appropriate services compared with those living in camp settings and the host population (IMC, 2019). Earlier research highlighted shortages of skilled mental health professionals, high turnover in MHPSS staff, inadequate training opportunities in mental health and limitations in the identification of mental health needs and referral (IMC and SIGI-JO, 2015; Mcnatt *et al.*, 2019; IMC, 2021). Inadequacies in the available services were a reported barrier to mental health service utilization amongst Syrian refugees in Jordan (IMC, 2017). Increasing psychosocial activities by non-specialized and specialized providers is recommended by the MHPSS working group in Jordan (IMC, 2021). This group is made up of representatives from UN agencies and NGOs who discuss the integration of different MHPSS services and ways for improving the referral between organizations.

### Study design, methods and process

This qualitative descriptive study used semi-structured individual and group interviews with people knowledgeable about PM+ and the mental health system for refugees in Jordan. In total, 17 individual interviews were done with PM+ facilitators (*n* = 5) and key informants (*n* = 12). Key informants (from now on called “informants”) involved those with expertise in PM+ (i.e. researchers, project workers, and supervisors on the STRENGTHS’ RCTs) and/or the Jordanian system (e.g. health professionals, policy advisors, and project managers). Additionally, four group interviews were conducted with PM+ participants (*n* = 20), i.e. Syrian refugees participating in the STRENGTHS’s RCTs. Each group contained five participants, with separate groups held for men and women, and those who completed at least four out of five PM+ sessions and those who completed less than four (former is seen as ‘completers’ and the latter as ‘non-completers’ in the RCTs).

Data collection took place from March 2020 to August 2021. Syrian refugees were approached by phone and informants by email. Informants were asked to recommend relevant people from their networks (snowball sampling). Interviews with PM+ participants and PM+ facilitators explored personal experiences with PM+ and views about reasons why some Syrians would choose not to attend PM+ sessions. Informant interviews explored perceptions on anticipated barriers and facilitators for scaling up PM+ in existing systems, including views on how to integrate PM+ and whom to involve. Interview guides were adapted for each sample, piloted and combined with interview questions for the process evaluations of PM+ RCTs to increase efficiency.

Group interviews were conducted face-to-face and individual interviews via video conferencing or telephone. Individual interviews with PM+ facilitators (average 20 min) and group interviews with PM+ participants (∼1.5 hours each) were conducted in Arabic by a local researcher (Jordanian clinical psychologist). Other informant interviews (average 57 min) were conducted in English by the lead author (health system researcher experienced in qualitative research and Dutch national). All individual interviews were audio recorded and transcribed verbatim. Group interviews could not be recorded due to camp restrictions; instead, note taking was used (a separate note taker was present during the interviews), and summary reports developed. Arabic interviews were translated into English. Ethical approval was received from the Jordan MoH and the authors’ institute. Written informed consent was obtained from all respondents.

### Data analysis

All pseudonymized data were analysed using qualitative data analysis software. Data were coded deductively, based on our conceptual framework (Woodward *et al.*, 2021), and inductively, with codes emerging from the data. Coding was first done independently by two authors and then recurrently discussed. Once the final coding framework was agreed, one author coded all the data and drafted the results.

## Results

Factors influencing the potential scale up (i.e. scalability) of PM+ in Jordan are presented according to our conceptual framework (Woodward *et al.*, 2021), starting with landscape developments, followed by considerations related to culture and structure. Scalability considerations presented here are based on the perceptions of interviewees about the expected interactions between the innovation (PM+) and potential adoptive systems in Jordan. Table 1 summarizes the key factors potentially enabling and/or constraining scaling up.

**Table 1.** Key factors enabling (+) and/or constraining (−) the potential for scaling up of PM+ in Jordan and related quotes

Factors influencing scalability	How factors enable (+) or constrain (−) scalability or both (±)	Selected quotes
*Landscape developments*		
COVID-19 pandemic	(−) Reduced physical access to target population (±) Remote MHPSS considered stigma reducing although there are physical access and privacy concerns (±) Increased openness and awareness about MHPSS; however, funding for MHPSS in competition with funding for COVID-19	“Otherwise I could add to the last point that while COVID might have contributed to a shift in public awareness towards more openness in speaking about MHPSS, an allocation of limited resources in staff and funds may rather prioritize public health concerns” Informant 12
Aid funding	(−) Reduced aid funding to the region (±) Political situation on the border of Jordan–Syria influences funding streams (−) Staff cuts in MHPSS due to reduced aid funding, which may impact PM+ implementation	“I think one of the big things that are happening is that a lot of the big money that goes in is aid money, or at least into this system where PM+ would be incorporated into, a lot of it is aid money. And I think I’ve heard some crazy statistics like there was 30% lower funding last year compared with the year before. 30% that’s [a lot]. Organizations were laying of 70–80 people in a day when that was announced” Informant 1
Political momentum	(+) Increasing awareness in government on the need for MHPSS (+) Mental health needs are an increasing political priority in the country	“There is a kind of appetite from the, from the government, again it’s more of kind of the bureaucracy, who’s taking the lead and all that. I think it easily can be implemented” Informant 5
*Culture*		
Approach to mental health	(−) Highly medicalized approach to mental health in government sector, with an emphasis on psychiatrists over other mental health providers (e.g. psychologists and nonspecialists) (−) Medication highly valued amongst patients, at the expense of psychological therapies (+) Biopsychosocial approach slowly gaining acceptance in state health system	“Sometimes our people [Jordanian/Middle Eastern] they do not believe in counselling, therapeutic sessions. They only believe in medication. Like “Give me medication then I will get improved”. So we have to change the mentality of our people first” Informant 3
Intervention modalities	(−) Group version of PM+ believed more (culturally) acceptable in Jordan compared with individual version (−) Face-to-face versions of PM+ (group or individual) perceived more effective than a possible digital version (+) Offering multiple modalities of PM+ (group, individual, and digital) considered to accommodate different beneficiaries	“Can it be online? So there are people honestly, who prefer this and who prefers that as it depends on the person himself. But for the category that we gave them which were the women, the mothers, I imagine that the best for them is not online, because usually they want to go out of the house, to see people, to talk to people[…]and it depends on the person himself; is (s)he busy, is (s)he available, how are his/her circumstances, does (s)he have an internet connection, all these things play a role”. PM+ facilitator 4
Stigma	(−) Mental health stigma as an obstacle for seeking MHPSS (+) Raising awareness about mental health, using non-stigmatizing language and limiting associations with ‘mental health’ (e.g. using unmarked mobile units) will enable PM+ implementation (+) Mental health stigma is slowly reducing, particularly in urban areas	“Stigma is still a big issue we are facing in our culture and in between refugees. Because of stigma, most of the people, maybe they didn’t seek any help until they reach big problems […] I think we can increase the awareness in the community and we will have to keep stigma in our mind; that it is still a problem. But I think in the last 10 years, stigma, as it is, become maybe a little bit not like before but for example, in the rural areas, it is still big issue”. Informant 6
Gender	(−) Women commonly not allowed to join groups without guardians’ approval (+) Offering childcare enables women to join PM+ sessions (+) Offering PM+ outside of traditional working hours enables men to join	“Why don’t you allow her [his wife] to go’. [he said] ‘We are very strict’. He is a very religious guy and he said ‘No, my wife is not allowed to go anywhere. Not only for your session, anywhere. The woman has to stay at her home and is not allowed to go outside”. Informant 4
*Structure*		
Target group and setting	(−) While relatively easy in a refugee camp, outside of camps, it is expected difficult to contact beneficiaries and/or to organize group sessions (e.g. transportation issue) (+) Expanding the intervention to Jordanian nationals was recommended as this may ease recruitment and make PM+ more attractive to potential funders	“First of all, how do you find Syrian refugees. It sounds obvious, and if you go to a refugee camp, it’s easy, but as soon as you step outside that camp, it’s impossibly hard, because there’s no register”. Informant 11
Policies	(−) Informants contradicted one another as to whether nonspecialist workers are legally allowed to provide psychological services (−) Labour policies limit the ability to employ Syrian refugees as PM+ facilitators	“It doesn’t really have a dedicated, you know, section or department. So Jordan has adapted a mental health and psychosocial support mental health action plan actually, recently, which is a very, very positive step, but in terms of rolling out this policy, it takes time and without a dedicated department on mental health, you know, things are not always followed up”. Informant 3
Human resources	(−) Distrust amongst Syrians and legal barriers in hiring Syrian refugees as PM+ facilitators (+) Jordanians accepted by Syrian refugees (RCT participants) as PM+ facilitators (+) Employing psychology graduates as PM+ facilitators may be beneficial (e.g. establishing trust and self-care) although other individuals may also be acceptable (i.e. positive attitude, good communication and sufficient training) (−) Retention of PM+ facilitators an issue, particularly amongst volunteers and those combing multiple roles (i.e. overburdening) (−) Limited number of qualified potential supervisors/trainers in the country	“[…]no human being is not affected, but as a person who studied Psychology or studied mental health[…]you are supposed to have like a training or a side that can help you, allows you to distinguish this from that. So I personally, like, no I didn’t get affected very much [by upsetting stories]’. PM+ facilitator 3 ‘…, for example in Jordan we only have one certified trainer in PM+. So obviously this needs to be expanded. You know we need more people; we need more local [PM+] trainers”. Informant 2
Financing	(+) Evidence of (cost)effectiveness for PM+ important for gaining support from potential funders (i.e. international donors and national government) (±) Financial and political support from MoH vital for sustainability of PM+ scale up although unlikely that Jordanian government has the ability to fully fund PM+ implementation	“I think showing the results is enough to bring funds[…]the Jordanian government should actually fund this thing. It will be reflected in a very positive way on people, and I think that there will be a very high turnout”. PM+ facilitator 3
Organizational structure	(−) Coordination of services for refugees is fragmented, involving different actors (NGOs, UN agencies and government), with gaps in communication and services; this could lead to disorganization and duplication of PM+ during scale up (+) Involvement of the government, particularly the MoH, and the MHPSS technical working group important to successful scale up (−) Limited number of qualified potential supervisors/trainers in the country challenges the organization of training and supervision of PM+ facilitators (+) PM+ ‘refresher’ trainings to upkeep quality of the intervention	“I’m emphasizing the necessity to provide this [PM+] in a coordinated manner’. Informant 10 ‘I am aware that this MHPSS technical working group is very active and one of their… [name given] […] who’s in the action plan, is really to strengthen this referral pathway […] who’s doing what where, for each organization which is regularly updated and that’s the strength of this MHPSS working group. I think it’s even shared online so that’s…I think is essential and this is what this MHPSS working group is good at, so…it can really play an active role on that”. Informant 9

INGO: international non-governmental organization.

### Landscape developments

Three developments at the wider context were identified as influencing the scalability of PM+, including the coronavirus disease 2019 (COVID-19) pandemic, aid funding and political momentum.

#### COVID-19 pandemic

Several informants noted that COVID-19–related restrictions impacted access to the target population in ongoing research on PM+ and other MHPSS initiatives. This was particularly true in camps, which heavily restricted access. The restrictions forced many organizations to provide their services online. While some informants reported that remote MHPSS delivery can reduce access barriers such as stigma and travel (i.e. anonymous access from home), a few also noted challenges in access to digital resources (e.g. smartphones) and privacy due to shared devices. Some informants observed increasing economic difficulties, including unemployment, among Syrian refugees and Jordanians, which further constrained access to digital resources and therefore the possibility of delivering PM+ remotely. Some informants commented that the pandemic resulted in MHPSS funds financially competing with funds to address COVID-19, which may negatively impact the scaling up of initiatives like PM+ (at least during a pandemic). However, several informants also noted that the pandemic shone a light on mental health needs, and policy-makers could thus be more engaged about integrating MHPSS interventions such as PM+.

#### Aid funding

Many informants believed that the feasibility of scaling up PM+ depends on the availability of aid funding. Some informants observed significant reductions in aid funding, mainly because major donors reduced funding to the region but also because of the changing political situation on the border of Jordan–Syria. These reductions led to staff cuts in the MHPSS sector in the Jordan, which limits the scalability of PM+ in the country, as there need to be sufficient staff for implementation, and enough capital to pay staff for training and facilitating PM+.

#### Political momentum

Several informants made it clear that the government was becoming more aware of the need for MHPSS services in the country. Reasons given were a recent sharp rise in suicides, along with the aftermath of other incidents such as a flooding disaster and the pandemic itself, which raised awareness within ministries of the need to provide MHPSS support. Several informants reported that it was an increasing priority for both governments and the general public to address MHPSS.

### Culture

Culture entails shared ways of thinking, mental models and perceptions. The approach to mental health, intervention modalities, stigma and gender were identified as the main scalability considerations in this category.

#### Approach to mental health

Some informants described the NGO landscape as being mostly made up of nonspecialists, while qualified mental health professionals dominated government-provided services. This difference in familiarity in task-sharing with nonspecialists may have been the reason why some informants thought it would be easier to scale up PM+ in the NGO sector. Several informants highlighted that a medicalized approach to mental health dominated the state health system, including an emphasis on psychiatrists over other mental health professionals. Additionally, on the demand side, the medicalized approach may be preferred, as two informants described patients expecting to receive medication and not understanding the benefit in psychosocial approaches to the problems they presented. Positively, two informants reported an increasing move towards a more biopsychosocial approach in addressing mental health issues in the government system although this was not yet widespread. According to several informants, PM+ could help fill a gap in the health system, but a cultural shift would need to take place to ensure acceptability.

#### Intervention modalities

There was a belief among PM+ researchers and project workers that offering PM+ in groups was more acceptable to Syrian refugees compared with individual or digital versions. Some PM+ participants highlighted that they enjoyed the group setting; however, when asked why other Syrians might choose not to participate, they mentioned the group element. A PM+ facilitator stated that, initially, participants were concerned others would share what they said during sessions, but once trust was established, they benefitted from the group setting. A PM+ researcher believed that an online modality may better address stigma, as well as transportation issues for participants outside of camps. Various interviewees, however, explained that digitally offering PM+ will be challenging as many refugees do not have access to reliable internet or smartphones (a barrier in infrastructure). Moreover, four out of five interviewed PM+ facilitators were convinced that only offering PM+ face-to-face would be effective. Overall, many interviewees believed that offering multiple modalities of PM+ was important, especially as different genders and age groups may respond differently to potential modalities.

#### Stigma

Stigma was described as a major issue for scaling up PM+ by nearly all informants although it was not explicitly discussed in group interviews with Syrian refugees. Some informants remarked that mental health stigma was slowly decreasing, particularly in urban areas. Some involved in the RCT noted that the recruitment of participants was easier when ‘psychological distress’, as opposed to ‘mental health’, was used to promote PM+. Several other informants similarly emphasized using non-stigmatizing language, such as ‘stress management’ and ‘problem-solving’, as a means to overcome stigma. Other suggestions made by informants for dealing with stigma were as follows: (1) having a confidential space for delivering PM+ (e.g. unmarked mobile units), (2) focusing on MHPSS awareness raising campaigns and (3) pitching groups as a way to help others to make participants look altruistic and avoid associating them with mental health disorders. Having a private space was considered by several informants and PM+ participants, especially important in camp settings, as most people know each other, and services and homes are closely situated.

#### Gender

Several informants, including PM+ project workers, reported that, for women, joining groups without a guardian’s approval could be challenging. One informant described a husband refusing his wife permission to join, and a similar situation was mentioned by a PM+ facilitator. Several informants further mentioned that offering childcare facilitated women joining PM+ sessions. This was echoed by PM+ facilitators and female PM+ participants who described caretaking duties as barriers for attendance. For men, two interviewees expressed the need to organize PM+ sessions outside of traditional working hours to enhance attendance.

### Structure

Structure is about how actors organize the things they do, either physically, institutionally or financially. The factors influencing the ways of organizing perceived important by interviewees for the scalability of PM+ are categorized as follows: target group and setting, policies, human resources, financing and organizational structure.

#### Target group and setting

Interviewees spoke about the feasibility of delivering PM+ inside and outside of camp settings. The recruitment of Syrian refugees during the RCT was, according to those involved, quite easy; the trial took place within a camp setting, meaning that the population was in one place and their movement restricted. Expanding the target population to include Jordanian nationals in addition to Syrian refugees was widely recommended amongst informants. Some explained that this would make reaching potential beneficiaries simpler (by widening the scope and not needing to target only Syrian refugees) and make the intervention more attractive to funders. That said, several interviewees believed that outside of camps the transportation of PM+ participants and PM+ facilitators to PM+ sessions may be logistically difficult.

#### Policies

Some informants contradicted one another on whether nonspecialists would be permitted to provide PM+ services in the government health system, which may have been because the legal framework surrounding those who can provide psychological services is rather new. One PM+ project worker insisted that anyone providing any kind of psychological service would need to be certified, while several other informants did not believe that there would be any legal barriers. One informant clarified that while the government was strict in terms of who could provide services within a clinical setting, PM+ would not be considered clinical and participants did not have psychiatric diagnoses, so there would be no legal barrier. In terms of whether Syrian refugees can legally be hired as PM+ facilitators, informants again disagreed on whether this was permissible. One informant involved in the RCT clarified that Syrian refugees have different regulations from Jordanians on how long they can work in the same job and how much they can be paid. For this reason, all PM+ facilitators in the trial were Jordanian.

#### Human resources

Primary human resources for the implementation of PM+ are licenced mental health specialists (i.e. PM+ trainers/supervisors), nonspecialists (i.e. PM+ facilitators) and one or more coordinators. Some informants were concerned about a lack of qualified potential supervisors due to the lack of mental health professionals in Jordan. As mentioned before under ‘policies’, Syrian refugees could not be hired as nonspecialists due to legal barriers, and therefore, PM+ facilitators in the trial were Jordanian. If these legal barriers are removed in the future, some informants and PM+ participants thought that it might still be beneficial to have Jordanian facilitators due to the general distrust between Syrians and positive experiences with Jordanian facilitators in the trials.

Several informants thought that hiring PM+ facilitators with bachelor’s degrees in psychology or similar subjects would be beneficial (as done in the PM+ definitive RCT). Some other informants believed the educational background of facilitators to be less important than having the right attitude, communication skills and sufficient training. A few informants explained that psychology graduates in particular face high unemployment, perhaps due to the limited budget of the MoH to employ them and the strict regulations regarding work in clinical settings. Therefore, there is a large pool of graduates who could be interested in working with PM+. Two interviewed PM+ facilitators described their backgrounds in psychology as helpful in gaining participant’s trust, and one in particular mentioned its usefulness for self-care when hearing the upsetting stories of participants.

It was clear from informants who worked on the RCT that PM+ facilitators needed to be both paid and dedicated to only providing PM+ to avoid losing them to other opportunities. Retention was reportedly a problem with volunteer facilitators for this reason. When NGO staff were facilitators (in the pilot RCT), two facilitators reported being overburdened as they juggled other duties simultaneously. Furthermore, as highlighted by several informants, due to general cuts in funding many organizations were already facing a shortage of staff, meaning that staff may already be overburdened and unable to take on additional duties. Two interviewees believed that having PM+ facilitators dedicated only to PM+ may prevent overburdening. However, it was also noted that a more limited workload likely reduces the revenue for the facilitator and could therefore still lead to retention issues.

#### Financing

Several informants explained that a large portion of the funding in Jordan for MHPSS services comes from international donors, with some additional funds provided by government ministries. Consequently, it was noted by several informants how the limited budget of the MoH meant it was unlikely it would fully fund the costs of providing PM+ services, such as staff salaries, supervision, training and travel. Evidence of effectiveness and cost-effectiveness was deemed important by multiple informants to secure both donor funding and government buy-in for the scaling up of PM+. Having support from the MoH, even if the majority of funds are from international donors, was described by some informants as important to long-term sustainability of scale up.

#### Organizational structure

The coordination of MHPSS services for refugees was described by several informants as fragmented, with NGOs and government bodies working together for some initiatives but with large gaps in communication and service provision. This fragmentation may challenge the scale up of PM+. Multiple informants therefore emphasized the need for clear coordination and effective collaboration between the many actors already offering MHPSS services in Jordan during scale up, including the need to avoid duplication of PM+ in the same areas. Many interviewees believed it important for PM+ to be regularly discussed during the monthly meetings of the MHPSS working group. The involvement of the government, particularly the MoH, was viewed by several informants as important to a successful scale up. Supplementary Figure 1 contains a map of potential stakeholders in the scaling up of PM+ in Jordan.

Many interviewees, including informants and facilitators, spoke about the importance of effective organization of training and supervision when scaling up PM+, particularly in light of a lack of qualified potential PM+ supervisors/trainers in the country. Besides initial PM+ training, some informants spoke about the need for refresher trainings in PM+. These were believed to support its quality and to be particularly important for staff whose role is not solely PM+ facilitation (and so may be less practiced in it).

## Discussion

This study examined the factors influencing the potential for scaling up a novel task-sharing psychological intervention for Syrian refugees in Jordan. Our findings were guided by key concepts of the ‘system innovation perspective’ (Woodward *et al.*, 2021), and we categorized scalability considerations as landscape developments, culture and structure. We identified various interrelated contextual and systemic factors that need to be considered when scaling up PM+ in existing systems in Jordan. These factors (and associated recommendations) may also be relevant for those involved in implementing similar task-sharing innovations in Jordan and other LMICs.

In line with the literature on scaling up task-sharing MHPSS innovations (Mendenhall *et al.*, 2014; Cohen and Yaeger, 2021; Troup *et al.*, 2021), our findings highlighted the importance of context (i.e. landscape). Positively, we found that there is political momentum to scale up mental healthcare in Jordan. More negatively, our interviewees predicted reduced donor funding and competing priorities (e.g. funding to address the COVID-19 pandemic). Likewise, previous research reported funding shortages for care and prevention of non-communicable diseases amongst Syrian refugees in Jordan (Akik *et al.*, 2019; Parmar *et al.*, 2021).

Our findings showed that the culture and structure of PM+ are more compatible with the culture and structure dominant in the NGO system compared with the government system in Jordan. The NGO system had fewer legal barriers surrounding the hiring of nonspecialists, and there was greater familiarity in task-sharing with nonspecialists. This means that scaling up PM+ through NGOs will likely be the scenario with the least resistance. Positively, there is a large overlap between these two systems in Jordan, particularly for vulnerable refugees in urban settings. This existing overlap may increase the feasibility of scaling up PM+ in urban areas and thereby its possible reach. Making PM+ first part of the services offered at NGO or combined NGO–government-run facilities may eventually open up doors for PM+ integration into solely government-run facilities and services.

Limited human and financial resources in the MHPSS system for Syrian refugees were identified in our study as key systemic barriers for scaling up PM+ in Jordan. While these resource challenges may create a demand for task-sharing interventions such as PM+ (because they do not require specialists and so are substantially cheaper and quicker to scale up), they also present serious obstacles, as similarly reported by reviews on MHPSS programmes in humanitarian settings and LMICs (Padmanathan and de Silva, 2013; Dickson and Bangpan, 2018; Troup *et al.*, 2021). In the long run, some level of system change is required to make it feasible to implement innovations like PM+ on a large scale and in a sustainable manner (i.e. integration into the governmental system), which is in accordance with the system innovation theory (Broerse and Grin, 2017). This need for a sustainability mindset is also highlighted by the latest results from the RCT of group PM+ in Jordan: the short-term benefits of the intervention (i.e. 3-month follow-up) were not sustained over longer periods of time (i.e. 12-month follow-up) (Bryant *et al.*, 2022b). The authors believed that ongoing stressors (partly due to COVID-19 pandemic) may explain the lack of sustained effectiveness and recommend booster sessions to remind participants of PM+ strategies and referral to more intensive treatment options for participants with more severe distress (i.e. stepped-care) (Bryant *et al.*, 2022b). Both recommendations require additional human and financial resources, which have been identified in our study as key systemic barriers to scaling up and are, therefore, difficult to achieve in the short term. In the following paragraphs, we propose three recommendations to address these resource barriers.

First, we recommend for PM+ to be integrated as part of psychology education in universities in Jordan. A training-of-trainers (ToT) model is an efficient way to rapidly train PM+ facilitators. Outside of a research setting, however, there is limited evidence on how ToT models for task-sharing psychological interventions can be sustainably funded and organized (Cohen and Yaeger, 2021). In the STRENGTHS RCT on group PM+ in Jordan (Bryant *et al.*, 2022a), psychology graduates served as effective group facilitators. If PM+ can be offered as part of psychology education in universities in Jordan, this may be a sustainable training model: all psychology graduates would then be trained in PM+, providing a large pool of possible PM+ facilitators, with PM+ training funded by the existing educational system.

Second, we recommend a co-financing model for the remuneration of PM+ staff during scale up. Staff include group facilitators, supervisors and one or more coordinators. Obtaining sustainable funding, however, can be difficult to achieve in LMICs like Jordan due to their high dependence on international aid: an often unstable, short-term and restricted funding source. Adding to this challenge, the Jordanian government had no fixed budget dedicated to mental health, and the available funds are commonly directed towards tertiary hospitals (IMC, 2017). Co-financing is a suitable model for LMICs, which would involve the government—thereby creating national ownership and strengthening the health system—and one or more financial partners (e.g. international, national and UN agencies and private investors). This model is already applied to MHPSS care for vulnerable refugees in urban areas in Jordan, with many health clinics co-financed by MoH and NGOs. Offering PM+ as a part of facilities where such co-financing already exists would be a logical way forward. In camp settings, however, there is a lack of existence of governmental services, meaning that other ways need to be sought to ensure sustainable funding.

Third, we recommend future research to focus on finding supervision models that work in LMICs. Our results indicate that the pool of potential supervisors is extremely limited in Jordan. Ideally, PM+ supervisors are skilled mental health professionals (Dawson *et al.*, 2015). However, there are severe shortages of such professionals in Jordan (IMC, 2017; WHO, 2022a). While less qualified staff could be resorted to, this may compromise intervention’ quality and effectiveness. Moreover, because nonspecialists are at risk of experiencing distress (Padmanathan and de Silva, 2013), it is important to have mental health professionals as PM+ supervisors as they are trained to detect such risks. More research is needed on finding the right balance between quality and local feasibility, including, as suggested by an interviewee, the possibility of ‘sharing’ supervisors between organizations implementing PM+. Additionally, it could be explored if finding supervisors outside of Jordan, but familiar with the context, is a feasible alternative. Such a supervision model would predominantly be at a distance, and therefore, its scalability will be dependent on users’ acceptability (i.e. some strongly prefer face-to-face formats) and the readiness of digital structures (of adopting systems, organizations and individuals). If alternative supervision models are used, these need to be assessed and monitored to ensure safety and quality of the intervention. We then recommend for recent work on the development of tools to measure and monitor the competencies of nonspecialist group facilitators (Pedersen *et al.*, 2021a; 2021b) to be extended to the competencies of their trainers/supervisors.

Aside from human and financial barriers, our study showed that there may be legal barriers that need to be overcome when integrating PM+ into the governmental system in Jordan. Interviewees expected this not to be the case for the NGO sector where nonspecialists are common and rules and regulations around licencing are limited. However, we argue that financial involvement and ownership of the government are desirable. This means that some level of integration into the governmental system is needed for a sustainable scale up, and therefore, the actors involved in PM+ implementation need to abide by existing rules, advocate for their change, or find creative solutions. Since interviewees in our study were not certain about the specifics on current regulations around hiring, licencing and remuneration of nonspecialists in existing systems in Jordan, this requires clarification before implementation.

Another key barrier to potential scale up raised in our study was stigma, highlighting the need for more action on stigma reduction in refugee communities. A recent Lancet commission recommends the involvement of people with lived experience of mental health conditions in anti-stigma programmes (Thornicroft *et al.*, 2022). Other studies have shown that more research is needed on the effectiveness of anti-stigma interventions tailored to refugees (Xin, 2020; Tahir *et al.*, 2022).

Finally, our findings give an indication of whom to involve (see stakeholder map in Supplementary Figure S1) and what actions to take to facilitate the scaling up of PM+ and similar task-sharing innovations. Since there is reportedly a lack of coordination between humanitarian actors and MoH in Jordan (Parmar *et al.*, 2021), the coordination needs to be a priority when scaling up PM+. Ideally, a national coordinator (person or organization) needs to be assigned who will oversee and support the implementation of PM+. This coordinator may also be responsible for lobbying such as ensuring that PM+ is mentioned in national health plans (e.g. JRP for the Syria crisis), setting up communities of practice where PM+ experiences can be shared amongst implementers, and communication with key stakeholders like the MHPSS technical working group to ensure smooth referral to PM+ and avoid duplication of efforts. Another recommended action is to expand PM+ in the country. This may involve expanding the target population to the host community and other Syrian refugees (in urban settings and teenagers), who also experience high proportions of mental health symptoms (IMC, 2017; Dehnel *et al.*, 2022). Expansion also includes offering various versions of PM+ (group/individual and face-to-face/hybrid). Our findings highlight the importance of using non-stigmatizing language when promoting and expanding psychological interventions like PM+. Such expansions require monitoring and evaluation. Political commitment expressed towards MHPSS will aid in gaining the political support for PM+ and requires maintenance during its expansion and integration.

Our study has several limitations. First, group interviews with Syrian refugees were limited by practicalities (i.e. audio recording was not possible; therefore, the analysis was based on summary reports). Second, we only interviewed Syrian refugees participating in the STRENGTHS’ RCTs and, therefore, those who sought and accepted MHPSS and were living in a camp setting. Third, individual interviews with PM+ facilitators were less in depth than planned for practical reasons (i.e. time restrictions and unreliable phone/online connection); this meant that some interview questions were not fully covered. Fourth, our study examined ‘potential’ factors in scaling but does not present an evaluation on ‘actual’ scaling up. A key strength of our study is that we were able to explore and combine the perceptions of different key actors (i.e. Syrian refugees, nonspecialist providers, informants with expertise in PM+ and the Jordanian system); this generated a rich understanding of the factors involved in scaling up psychological innovations in Jordan.

## Conclusions

From this study, we can conclude that various interrelated contextual and systemic factors are likely at play when scaling up novel task-sharing psychological interventions like PM+ in Jordan. Knowledge gained from this scalability study may inform the development of a scaling-up strategy. The political momentum in Jordan needs to be utilized, and sustainable funding made available for staff, training, supervision, infrastructure, coordination, expansion and evaluation of ‘actual’ scaling up of PM+. Initially, funding and implementation of PM+ may be done through NGOs—which is already happening in Jordan—but eventually, increased ownership and support of the national government are needed if task-sharing approaches are to be sustainable and transformative. The integration of task-sharing psychological innovations has the potential to positively change mental health systems, making them more community based and therefore accessible. Such a system innovation could benefit both refugees and host communities in LMICs, which is in light of the large mental healthcare gap urgently needed.

## Supplementary Material

czad003_Supp

## Data Availability

All relevant data used to reach the conclusions drawn in the study are within the paper.

## Abbreviations

COVID-19 = coronavirus disease 2019

LMIC = low- and middle-income country

MHPSS = mental health and psychosocial support

MoH = Ministry of Health

NGO = non-governmental organization

PM+ = Problem Management Plus

RCT = randomized controlled trial

STRENGTHS = Syrian REfuGees MeNTal HealTH Care Systems

UN = United Nations

## Reflexivity statement

The authors include a mixture of men and women and include various levels of academic seniority. The majority of authors are living in high-income countries (the Netherlands, the UK and Australia) and two authors are from Jordan. The authors cover a variety of research expertise, including global health, mental health, health systems and conflict-affected populations.
