# Supplementary material for: Scaling up task-sharing psychological interventions for refugees in Jordan: a qualitative study on the potential barriers and facilitators
Source: Health Policy Plan. 2023 Jan 12;38(3):310–20. doi: 10.1093/heapol/czad003 (PMC10019561; doi:10.1093/heapol/czad003)
Supplement: czad003_Supp [file czad003_supp.zip › Supplementary Figure 1.docx]

**Supplementary Figure 1: Stakeholder map**

Stakeholders are actors (individuals or groups) that can affect, or can be affected by, the potential integration of novel psychological interventions like PM+. Many possible stakeholders were identified during the interviews and are displayed in the stakeholder map in Figure 1. Stakeholders were grouped in seven different categories, including government, foundations, academic community, healthcare workers, beneficiaries, donors, and (international) NGOs. This map is not exhaustive but serves as a starting point for those involved in the PM+ scaling up strategy formulation and implementation in Jordan.


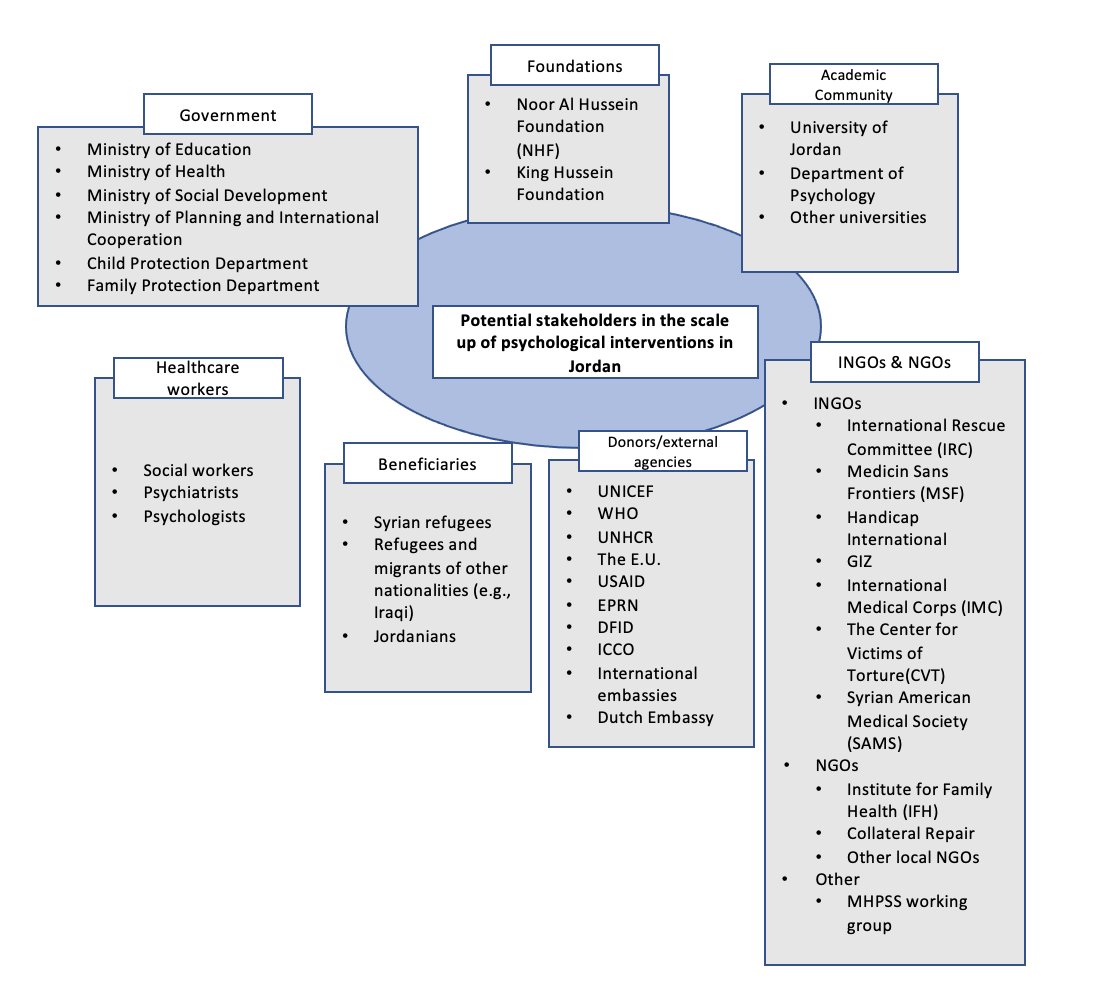


**Figure 1.** Stakeholder map
